# Development of conducting paper-based electrochemical biosensor for procalcitonin detection

**DOI:** 10.5599/admet.1575

**Published:** 2023-01-30

**Authors:** Yachana Gupta, Aditya Sharma Ghrera

**Affiliations:** Applied Science Department, The NorthCap University, HUDA-Sector 23A, Gurugram, India

**Keywords:** Gold nanoparticles, PEDOT, PEDOT:PSS, procalcitonin, monoclonal antibodies, chronoamperometry

## Abstract

In the present research, an advanced cellulose fiber paper (CFP) based biosensor is developed. This sensor is modified with nanocomposites containing poly(3,4-ethylene dioxythiophene) polystyrene sulfonate (PEDOT:PSS) as the main matrix and functionalized gold nanoparticles (PEDOT:PSS-AuNP@CFP) for the selective and sensitive detection of bacterial infection (BI)-specific biomarker procalcitonin (PCT). Scanning electronic microscopy, Fourier transform infrared spectroscopy, and X-ray diffraction are used to characterize the PEDOT:PSS-AuNP nanocomposite. This biosensor exhibits a high sensitivity of 1.34 μA (pg mL^-1^)^-1^ in the linear detection ranges of 1-20×10^4^ pg mL^-1^, and a 24-day life span for PCT antigen detection. Anti-PCT antigenic protein is used for immobilization for PCT quantification. The results of electrochemical response studies showed that this conductive paper bioelectrode had good reproducibility, stability, and sensitivity in physiological ranges (1-20×10^4^ pg mL^-1^). Further, the proposed bioelectrode is an alternative choice for point-of-care PCT detection.

## Introduction

Paper is excellent as a basis for manufacturing analytical sensors due to its flexibility, disposability, copiousness, and minimal cost [1-3]. The cellulose fiber paper (CFP) is strong, has a very good bonding ability, and its application has specific demands. Due to its good absorption capacity, particles can be retained on the surface for a longer time, and as a substrate, it exhibits strong antigen-antibody interaction [4,5]. This CFP substrate is used in the development of various sensors and other research applications at the nanoscale, such as membrane affinity, tissue engineering, and assay development, because it has a large surface area, one-dimensional configuration, and good coordination with surface molecules [6-8]. Currently, research is focused on improving the conductivity of paper substrates compared to other conventional substrates such as ceramics and glass. Conducting polymers such as polyaniline [9,10], polypyrrole [11], poly(phenylenevinylene) [12], polyacetylene [13], polythiophene [14], poly(para-phenylene) [15], poly(3,4-ethylene dioxythiophene) polystyrene sulfonate (PEDOT:PSS) [16,17] and polyfuran [18], *etc.* are used for surface modification. Among the several conducting polymers, PEDOT:PSS is the most appropriate and multipurpose due to its advantages of being film-forming ability, good thermal stability, homogeneous set-up on paper, mechanical flexibility, and exhibiting remarkable electrochemical properties in the development of paper-based biosensors [19-22]. In addition, solvent treatment of PEDOT:PSS can promote rapid electron transfer between the paper surface and the solution, thereby increasing conductivity. Conductive polymers, when integrated with gold nanoparticles (AuNP), silver, and other metals and their oxide nanoparticles, etc., can improve electrochemical, optical, and physical properties [23-25]. Various studies have used nanoparticles with PEDOT:PSS to improve surface properties and electrical conductivity [26-28]. Several other electrochemical biosensor sensors have been established for the detection of bacterial infection (BI)-specific biomarker procalcitonin (PCT) [29-32].

PCT is a biomarker specific for BI detection and has gained much attention as a potential solution to the problems associated with determining appropriate antibiotic use [33]. It has a molecular weight of 13 kDa and is composed of 116 amino acid proteins. Thyroid C cells produce the PCT, which is then converted to calcitonin before entering the bloodstream. In children, PCT levels can rise under any circumstances. PCT is rarely detected in detectable quantities in the blood of healthy individuals. According to numerous studies, serum PCT levels rise in individuals with sepsis and reduce after a successful diagnosis of BI with an antibiotic. Thus, the quantitative assessment of PCT levels earlier and afterward BI diagnosis may contribute to the early detection of this malignant infection. A variety of conductive polymer-modified electrochemical sensors have been reported for PCT detection [34-38], but these approaches suffer from limitations due to multifaceted pre-treatment steps and the use of exorbitant electrodes. As a result, a facile, profligate, flexible, and economical substrate is required for PCT detection.

In the present study, an electrochemical biosensor is developed for the detection of BI-specific biomarker PCT using CFP as the electrode platform, and for the first time, a composite of AuNP and conductive polymer (PEDOT:PSS) is proposed for coating the cellulose fiber paper and for the signal amplification. The dip coating method is used to fabricate cellulose paper substrate with PEDOT:PSS-AuNP composite. In this method, a composite of PEDOT:PSS and AuNP was prepared to increase the electrical conductivity of the paper electrode. The developed CFP-based sensor is inexpensive and biodegradable compared to other expensive substrates such as glassy carbon electrodes, ITO, FTO, *etc.* On the other hand, paper-based methods are being developed because they have various advantages such as affordability, are disposable, and require a small sample volume. PEDOT:PSS-AuNP@CFP shows good antibody-antigen interaction with a low detection limit of 1 pg mL^-1^ and sensitivity of 1.34 μA (pg mL^-1^)^-1^ in linear ranges of 1-20×10^4^ pg mL^-1^, respectively. This bioelectrode also shows good reproducibility and improved stability. In this research, a cost-effective, user-friendly, portable, and disposable PEDOT:PSS-AuNP composite-modified CFP-based biosensor is evaluated as a new approach for PCT detection. Using the chronoamperometry method, efforts were made to achieve a satisfactory response of the developed biosensor.

## Experimental

### Required materials and apparatus

PEDOT:PSS was purchased from Sigma Aldrich, and ethylene glycol was purchased from CDH chemicals. Bovine albumin serum (BSA), auric chloride, trisodium citrate, monobasic sodium phosphate, and disodium phosphate were purchased from CDH Chemicals. Tween-80 was purchased from Thomas Baker (Chemicals). Monoclonal antibody (Mab) was purchased from www.mybiosource.com. Cellulose fiber paper (21×260mm) was procured with advanced micro devices (MDI) membrane. For electrochemical characterization, a three-electrode Autolab PGSTAT204 potentiostat/galvanostat (Eco Chemie, Netherlands) was employed. In 0.1 M KCl containing 10 mM [Fe(CN)_6_]^3-/4-^, a cellulose fiber paper strip was applied as a working electrode, a platinum foil as a counter electrode, and Ag/AgCl as a reference electrode. Field emission scanning electron microscope (FESEM) model A JEOL JSM-7610F plus was used to investigate the nanocomposite morphology. To ensure that the PEDOT:PSS-AuNP composite is formed, Fourier transform infrared spectroscopy (FTIR) (model-Bruka ALPHA) and X-ray diffraction (XRD) (model-Rigaku Smart lab studio) techniques were used. A low current source and a digital microvoltmeter (model: DMV 1001) were used to assess conductivity using four-probe techniques (model: LCS 102).

### Synthesis of PEDOT-AuNP nanocomposite

A colloid solution of spherical-sized AuNP was prepared by the earlier-mentioned citrate method [39]. Before the synthesis of PEDOT:PSS-AuNP nanocomposite, an aqueous suspension of 2.4 wt% PEDOT:PSS and 5 % of ethylene glycol were prepared and stirred for 30 minutes. After that, this suspension was mixed with a colloidal solution of AuNP (ratio 1:1) and left for ultrasonication for 1 h. The prepared PEDOT:PSS-AuNP composite was kept at 4 °C for further use**.**

### Fabrication of CFP electrode

Before fabricating the CFP electrodes, CFP was cut into a (1×1 cm^2^) dimension and washed three times in ethanol and deionized water. Thereafter, Tween 80 surfactant was used for stabilization and dried at 37 °C for 24 hours. These stabilized electrodes were dipped in a PEDOT:PSS-AuNP composite previously synthesized. The PEDOT:PSS-AuNP with CFP electrodes were kept for ultrasonication for 40 minutes. The electrode's color change from white to black indicates that PEDOT: PSS-AuNP composite has been deposited on the paper. After that, the electrodes were dried in a hot air oven at 40 °C for 30 minutes. The electrodes that resulted were stored in the freezer for future use. The PEDOT:PSS-AuNP@CFP electrode is now referred to as a CP (conducting paper) electrode.

### Antibody immobilization

**Figure 1.**
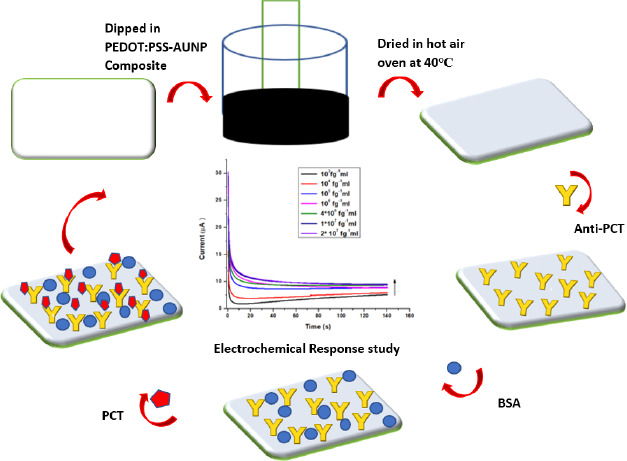
Schematic diagram of the fabrication of conducting paper-based PCT biosensor.

To immobilize the antibody onto the CP electrode, a monoclonal antibody (anti-PCT) which is specific to PCT was used. 10 μl of 0.08 mg mL^-1^ concentration of anti-PCT in PBS (7.4) was dropped at the CP electrode and dried for 30 minutes. After that, 10 μl of 0.25 % blocking agent BSA was dropped over the CP electrode to block the unspecific sites. Thereafter it was kept in the undisturbed position for 30 minutes. Now, a prepared BSA/anti-PCT/CP electrode was used for the electrochemical response studies. For the electrochemical response studies, 1 cm of prepared BSA/anti-PCT/CP was dipped into a redox probe solution. To ensure the anti-PCT immobilization on to CP surface, electrochemical characterization techniques and spectroscopic techniques were involved. The fabricated PCT/ BSA/anti-PCT/CP schematic has been demonstrated in Figure 1.

## Results and discussion

### Spectroscopic characterization

**Figure 2.**
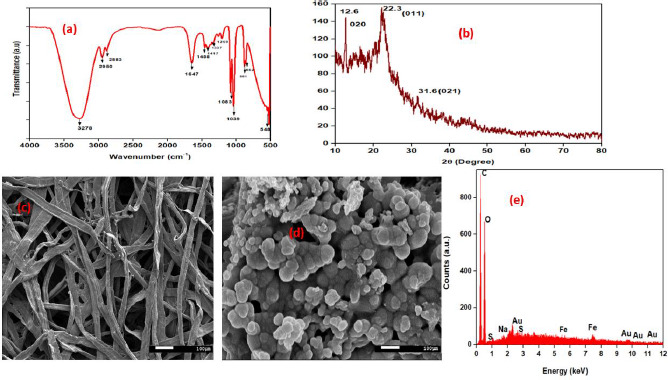
(a) and (b) FTIR and XRD spectra of PEDOT:PSS-AuNP, (c) FESEM image of bare CFP substrate, (d) FESEM image of CP, and (e) EDAX analytical performance of CP.

Pre-prepared PEDOT:PSS-AuNP nanocomposite solution was analyzed through FTIR measuring instrument. Figures 2a and 2b depict FTIR and XRD spectra of PEDOT:PSS-AuNP nanocomposite films. FTIR spectroscopy provides data at the microscopic level, i.e., the changes that take place in the PEDOT:PSS backbone as a result of the existence of charged nanoparticles in their surroundings. PEDOT:PSS-AuNP has wide-ranging and strong absorption peaks in the FTIR spectrum at 3276, 2952, and 2884 cm^-1^ [40], respectively, correlating to the asymmetric stretching of hydroxyl (–OH), carbonyl (–C = O), and epoxy (C-O) groups [41]. The C–C stretching from PEDOT's thiophene ring is indicated by the peaks at 1337 cm^−1^ and 1416 cm^−1^ [42]. The peaks at 1646 and 1458 cm^−1^ are associated with C-C stretching in PSS aromatic rings, respectively. The C–S stretching of the thiophene ring in PEDOT coincides with the additional peaks at 882, 669, 569, and 516 cm^−1^ [42,43]. Peak shifts in FTIR bands are caused by nanoparticles as a result of signal transduction in both the PEDOT and PSS chains. When charged nanoparticles appear, electrostatic attraction between nanoparticles and PSS occurs, reducing the interaction between PEDOT and PSS. As a result, the validation of PEDOT and PSS chains differs, resulting in improved connectivity. The crystalline phase of the developed PEDOT:PSS-AuNP composite was investigated using the X-ray diffraction (XRD) technique, and the resulting XRD patterns are displayed in Figure 2. PEDOT:PSS-AuNP composite exhibited three distinct peaks at 2AAAAAAAAAA = 12.6°, 22.3°, and 31.6°. All three peaks corresponded to standard Bragg reflections (020), (011), (021), and monoclinic cubic lattice. Because it follows seven crystal system a ≠ b ≠ c, α = γ = β ≠90°. The considerable diffraction at the 22.3° peak implies that zero-valent gold's optimal growth configuration was set to (011) [44]. Solids with a molecular scale and a 3D pattern of atoms or molecules with an average crystal size of 0.371 nm are referred to as molecular solids [43].

FESEM measurements demonstrate the morphologies and structural features of the as-synthesized PEDOT:PSS-AuNP nanocomposite. The CFP substrate was properly cleaned and dried several times. After that, the PEDOT: PSS-AuNP solution was dropped at the CFP surface and dried. The coating of CFP with the PEDOT: PSS-AuNP solution was performed four times. After the coated CFP substrate had been completely dried, it was used for FESEM characterization. The SEM image of bare cellulose fiber paper substrate and prepared PEDOT:PSS-AuNP nanocomposite is shown in Figures 2c and 2d, respectively. Through the FESEM, it was found that PEDOT:PSS-AuNP nanocomposite was successfully deposited onto the CFP substrate and can be used for further studies. The Energy dispersive X-ray analysis (EDAX) spectra of AuNPs incorporated PEDOT:PSS composite is shown in Figure. 2e. The EDAX spectra and elemental mapping of the FESEM images show that AuNPs are distributed uniformly in the PEDOT:PSS composite film. The results of the EDAX analysis of PEDOT: PSS-AuNP are shown in Table. 1. The EDAX spectrum of pelletized PEDOT:PSS-AuNP with a carbon and oxygen peak is shown in Figure 2(e), and other peaks in the spectrum correspond to sodium, sulphur, iron, and gold. Sulphur and iron were present as a result of the use of PSS and sodium due to citrate as oxidizing agents. AuNP corresponds to Au. In Table 1 the C:O mass ratios in PEDOT:PSS-AuNP films were estimated to be 42.6 and 41.3 %, respectively. The atom ratios of C: O. PEDOT:PSS-AuNP composite films were estimated to be 56.2 and 40.9 %, respectively.

### Electrochemical characterization

Electrochemical impedance spectroscopy (EIS), cyclic voltammetry (CV), and chronoamperometry were used to characterize the CP electrode. 10 mM [Fe(CN)_6_]^3-/4-^ with 0.1 M KCl solution was used to monitor changes at each step.

**Figure 3.**
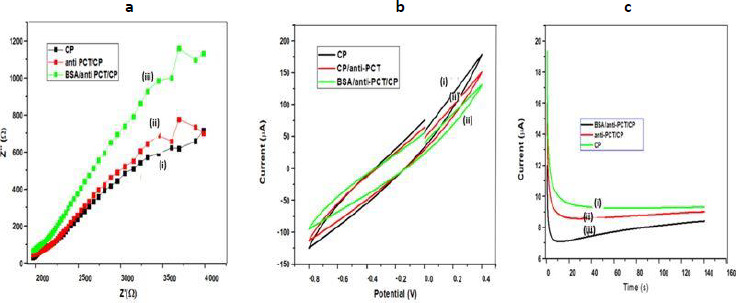
Electrochemical (a) EIS, (b) CV and, (c) chronoamperometry studies of unmodified and modified bioelectrodes

EIS is an effective approach for determining the intercalation resistance at the conductive interface and investigating electrodes with surface functionalization [45-47]. Figure 3a displays Nyquist plots of several modified electrodes, generated by graphing the imaginary part (*Z*′′) on the y-axis and the real component (*Z*′) on the x-axis. Usually, the charge transfer resistance (*R*_ct_) at the electrode surface is determined by the Nyquist plot of the diameter of the semicircle part, which indicates the electron transfer kinetics at the interface. Due to the large impedances and complex features of the impedance spectra shown in Figure 3a, Rct could not be accurately determined but roughly estimated by the non-linear least square fitting of the experimental results assuming Randles circuit [*R*_s_(*R*_ct_*C*_dl_)] of the electrochemical cell.The alteration of the electrode surface was revealed by the decrease or rise in *R*_ct_ values. The *R*_ct_ value of CP was roughly estimated to be 4220 Ω (curve i), and after immobilization of anti-PCT/CP, to 4335 Ω, which is higher than the CP electrode. When the blocking agent was used *R*_ct_ value of BSA/anti-PCT/CP further increased and reached to 4400 Ω [48-50]. This could be due to BSA's insulating properties, which prevented electron transport at the electrode-electrolyte interface. All of these comparative fluctuations in the *R*_ct_ values of dissimilar electrodes support electrode modification.

The electrochemical behavior of the designed bioelectrode (BSA/anti-PCT/CP) with varying PCT concentrations was also observed using the cyclic voltammetry (CV) technique in the potential range of -0.8 to 0.4 V and at a scan rate of 50 mV s^-1^ (Figure 3b). CV is an efficient and important method for visual observation of surface morphology and analyzing the integrations of electrode materials [51]. As a result, CV was selected to examine the modification in electrode behavior caused by electrode modification. Figure 3b demonstrates the CV of (i) CP, (ii) anti-PCT/CP, and (iii) BSA/anti-PCT/CP. All CVs exhibit resistive behavior without current peaks at 50 mVs^-1^, indicating no fast charge transfer occurs in the system. The highest currents were registered on bare CP material (curve i). Curve (ii) shows a decrease in the current values, which may be attributed to the fact that antibodies have been successfully immobilized on CP. The antigen-antibody complexes so formed have produced additional barriers and prevent electron exchange between the working electrode and the solution, thereby decreasing the peak current values [52]. The currents of the BSA/anti-PCT/CP bioelectrode is even lower after modification with shielding BSA as the obstructing agent for the non-specific signaling pathways. As a result of its electrical insulation, BSA hinders the transfer of electrons between the electrode and medium, resulting in a reduction in peak current value [52]. All of these variabilities in peak current value assist and verify the fabrication of electrodes with biomolecules.

The chronoamperometric approach was used to analyze the electrochemical behavior of (i) CP, (ii) anti-PCT/CP, and (iii) BSA/anti-PCT/CP bioelectrodes at a potential of 0.20 V. The chronoamperometric plot for different modifications on CFP electrodes is shown in Figure 3c and it was found that for all electrodes after the initial transient period of about 10 s, the current assumed a steady value. For the particular instance of solution-processed CP, the current was 9.7 μA [(curve (i)]. Due to the macromolecule size of anti-PCT, there was a slight decrease in current for the anti-PCT/CP electrode (8.7 μA; curve ii). Furthermore, for BSA/anti-PCT/CP, decreases in electrochemical current (5.7 μA; curve iii) were observed. This is due to BSA's insulating properties, which impeded the electron transference mediator's entry into the substrate.

### Electrochemical response studies

**Figure 4.**
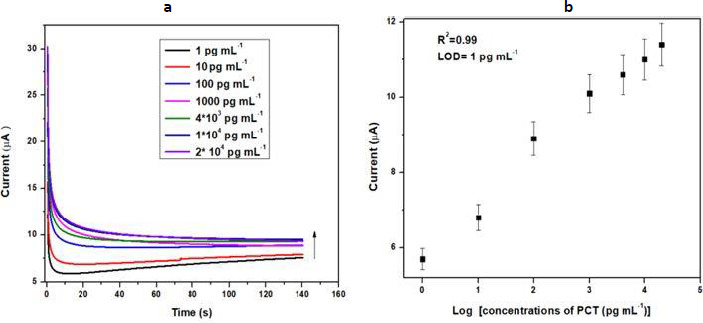
**(a)** Chronoamperometry response study of PCT/ BSA/anti-PCT/CP bioelectrode in 10 mM [Fe(CN)_6_]^3-/4-^redox probe with 0.1 M KCl solution; **(b)** calibration plot of the bioelectrode between the response current and PCT concentrations*.*

Chronoamperometry is also known as potentiostatic coulometry. A steady voltage is given to the working electrode throughout this procedure, and the current is measured over time. The analyte is reduced or oxidized at a distinct oxidation potential. Chronoamperometry experiments were conducted to evaluate the physical transformation per mole of electron flow at the electrode-electrolyte interaction over a brief time span at a constant potential. The minimum detection potential 0.20 V was used to produce electrochemical response. The electrochemical response of the BSA/anti-PCT/CP bioelectrode was measured in 20 ml of 10 mM [Fe(CN)_6_]^3-/4-^ in 0.1 M KCl solution for various concentrations of PCT (1-20 ×10^4^ pg mL^-1^) over a ten-minute incubation time. The chronoamperometry was conducted at 0.20 V for 140 seconds to ensure the antibody-antigen interaction was completed. Figure 4a demonstrates the difference in the response current as a function of PCT concentration (1-20×10^4^ pg mL^-1^). For this objective, the BSA/anti-PCT/CP bioelectrode was estimated with 10 μL of PCT solution and it was found that when PCT concentration rises, the response current also increases [6,53]. The calibration plot (Figure 4b) was constructed in the 1-20×10^4^ pg mL^-1^ ranges, which was produced by displaying the PCT concentration *vs.* response current at 140 s. From this plot, it was observed that the response current is dependent on concentration. Equation (1) describes how the current varies with concentration. The LOD of the developed sensor was found to be 10^3^ fg mL^-1^, and the linear correlation resulted in a regression coefficient of 0.99.


(1) *I* = 1.34 *c*_PCT_ + 1.72

### Reproducibility and stability

**Figure 5.**
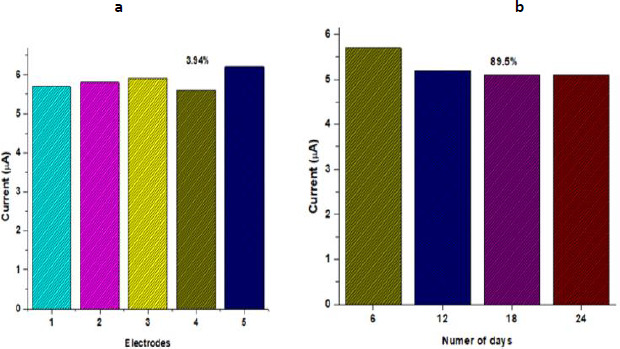
**(a)** Reproducibility **(b)** stability of the BSA/anti-PCT/CP in 0.1 M KCl containing 10 mM [Fe(CN)_6_]^3-/ 4-^

To evaluate the reproducibility of the BSA/anti-PCT/CP bioelectrode, five distinct electrodes were fabricated under the same optimized condition, and the biosensing characteristics were assessed using chronoamperometry. Figure 5a demonstrates that there was a minimal difference in current for the five distinct electrodes, with a relative standard deviation of 3.94 % (mean value 5.84), implying that the BSA/anti-PCT/CP bioelectrode had good reproducibility. The assessment of the developed PCT biosensor's long-term stability is essential to the biosensor's applicability for practical applications. The response current of the fabricated BSA/anti-PCT/CP bioelectrode was measured every six days to study the stability of the biosensor. The fabricated BSA/anti-PCT/CP bioelectrode electrode maintained approximately 89.5 % of the initial current response after 24 days, demonstrating that the BSA/anti-PCT/CP bioelectrode has good stability (Figure 5b). This improved stability is due to AuNP/CFP's strong affinity for PEDOT:PSS, which is presented as a result of repeated pre-treatment. In comparison to previous biosensors for PCT detection, this related work demonstrated (Table 2) PEDOT:PSS:AuNP composite fabricated CFP substrate exhibited promising detection range, sensitivity, and enhanced shelf life

### Electrical conductivity studies

The four-probe method was used to evaluate the conductivity of CF paper by employing a digital microvoltmeter and a low-current source. Each modification step is measured to explore the conductivity of CF paper electrodes. The conductivity value of AuNP coated on CF paper was 3.45×10^-5^ S cm^-1^, while the conductivity value of PEDOT:PSS-ethylene glycol suspension coated CF paper was 2.57×10^-4^ S cm^-1^. The zero-overlap semimetal structure of PEDOT:PSS, which contains electrons and holes as charge carriers, accounts for its high electrical conductivity. Furthermore, the PEDOT:PSS-AuNP composite was coated on paper to increase conductivity, which was found to be 1.09×10^-3^ S cm^-1^. PEDOT:PSS-AuNP can facilitate electron transfer between electroactive species and electrode substrates, indicating that the integrated platform can potentially further promote bioelectrode conductivity.

## Conclusion

The present article describes a facile strategy to detect BI-specific biomarker PCT. The PEDOT:PSS-AuNP nanocomposite was used to fabricate a CFP-based electrochemical sensor. For the deposition of PEDOT:PSS/AuNP, CFP substrate is considered because of its good absorption capacity, particle retaining properties, cost-effectiveness, portable, disposable, and user friendly. Modifications in the CFP surface were investigated using electrochemical characterization. Deposition of PEDOT:PSS-AuNP composite on the CFP surface was studied using FESEM and EDAX spectroscopy. Through the XRD analysis, crystalline size was monitored and with FTIR studies presence of functional groups was investigated. Synergistic interaction of AuNP with PEDOT:PSS could lead to significantly improved sensitivity and lower electrode overpotentials. Additionally, the PEDOT:PSS/AuNP modified CFP shows enhanced electrochemical properties rather than unmodified electrodes. The modified PCT/BSA/anti-PCT/CP bioelectrode demonstrated an excellent detection limit of detection (1 pg mL^-1^) with a linear detection range (1-20×10^4^ pg mL^-1^). A-prepared bioelectrode exhibits excellent reproducibility and high stability for at least four weeks, with negligible change in electrochemical response. Furthermore, A sensor of this type may also be realistically designed for real-time sample analysis with adequate sensitivity and efficiency. This implies that our well-known electrochemical biosensor would be ideal for bioanalytical research.

## Figures and Tables

**Table 1.** Results for EDAX analysis of CP electrode

Element	Line	Content, wt.%	Content, at.%
C	K	42.6	56.2
O	K	41.3	40.9
Na	K	1.4	0.9
S	K	1.2	0.6
Fe	K	1.3	0.4
Au	L	12.3	0.1

**Table 2.** Comparison of the electrochemical sensing performance of different nanocomposites modified electrodes towards PCT detection.

Modified electrode	Substrate	Detection method	Concentration, pg mL^-1^		Ref.
			Linear range	Detection limit	
G-Co@ NCNBs	GCE	One-pot solvothermal strategy and self-catalyzed chemical vapor deposition	0.1 ~ 100,000	0.01	[54]
rGO-Au	GCE	Electrodeposition	1.00 to 20,000	0.43	[29]
PANI NRs/rGO-Au	GCE	Electrochemiluminescence	0.10 to 50,000	54,000	[55]
GC/MWCNTs, CS, GA	GCE	Electrochemical immunoassay	10 to 350,000	0.5	[56]
PEDOT:PSS-AuNP	Paper	Dip coating	1 to 200,000	1	This work
